# Mechanisms of metal-dependent non-redox decarboxylases from quantum chemical calculations

**DOI:** 10.1016/j.csbj.2021.05.044

**Published:** 2021-05-26

**Authors:** Xiang Sheng, Fahmi Himo

**Affiliations:** a National Engineering Laboratory for Industrial Enzymes and Tianjin Engineering Research Center of Biocatalytic Technology, Tianjin Institute of Industrial Biotechnology, Chinese Academy of Sciences, and National Technology Innovation Center for Synthetic Biology, Tianjin 300308, PR China; b Department of Organic Chemistry, Arrhenius Laboratory, Stockholm University, SE-10691 Stockholm, Sweden

**Keywords:** AHS, amidohydrolase superfamily, LigW, 5‐carboxyvanillate decarboxylase, γ-RSD, γ‐resorcylate decarboxylase, γ-RS, γ-resorcylate, 2,6-DHBD, 2,6‐dihydroxybenzoic acid decarboxylase, 2,3-DHBD, 2,3‐dihydroxybenzoic acid decarboxylase, IDCase, *iso*-orotate decarboxylase, 5-CV, 5-carboxyvanillate, 5-NV, 5-nitrovanillate, MIMS, membrane inlet mass spectrometry, 2-NR, 2-nitroresorcinol, 5caU, 5-carboxyuracil, QM/MM, quantum mechanics/molecular mechanics, Biocatalysis, Decarboxylase, Reaction mechanism, Density functional theory, Transition state

## Abstract

Quantum chemical calculations are today an extremely valuable tool for studying enzymatic reaction mechanisms. In this mini-review, we summarize our recent work on several metal-dependent decarboxylases, where we used the so-called cluster approach to decipher the details of the reaction mechanisms, including elucidation of the identity of the metal cofactors and the origins of substrate specificity.

Decarboxylases are of growing potential for biocatalytic applications, as they can be used in the synthesis of novel compounds of, *e.g.*, pharmaceutical interest. They can also be employed in the reverse direction, providing a strategy to synthesize value‐added chemicals by CO_2_ fixation. A number of non-redox metal-dependent decarboxylases from the amidohydrolase superfamily have been demonstrated to have promiscuous carboxylation activities and have attracted great attention in the recent years. The computational mechanistic studies provide insights that are important for the further modification and utilization of these enzymes in industrial processes. The discussed enzymes are: 5‐carboxyvanillate decarboxylase, γ‐resorcylate decarboxylase, 2,3‐dihydroxybenzoic acid decarboxylase, and *iso*-orotate decarboxylase.

## 1 Introduction

Decarboxylases are increasingly used in organic synthesis for the preparation of novel compounds, for example optically pure drug molecules and high-value chemicals from renewable resources [1], [2], [3], [4]. In addition to their natural decarboxylation reactions, many decarboxylases have been demonstrated to have promiscuous activities, catalyzing *e.g.* C–C bond formation [5], [6], hydration [7] and racemization reactions [8], further increasing their potential applications in industrial processes. Among these activities, a particularly interesting one is the promotion of carboxylation reactions, i.e. the reverse direction of the natural reaction. This has attracted a lot of attention in recent years, as it provides a biocatalytic strategy to synthesize value‐added chemicals using CO_2_ as a C1‐building block.[9], [10], [11], [12] For example, a number of metal-dependent decarboxylases have been established to enable the carboxylation of aromatic substrates [13], [14], [15], [16], representing thereby a sustainable alternative to the traditional Kolbe–Schmitt process that usually shows low regioselectivity and requires harsh conditions in terms of pressure and temperature [17].

**Scheme 1:**
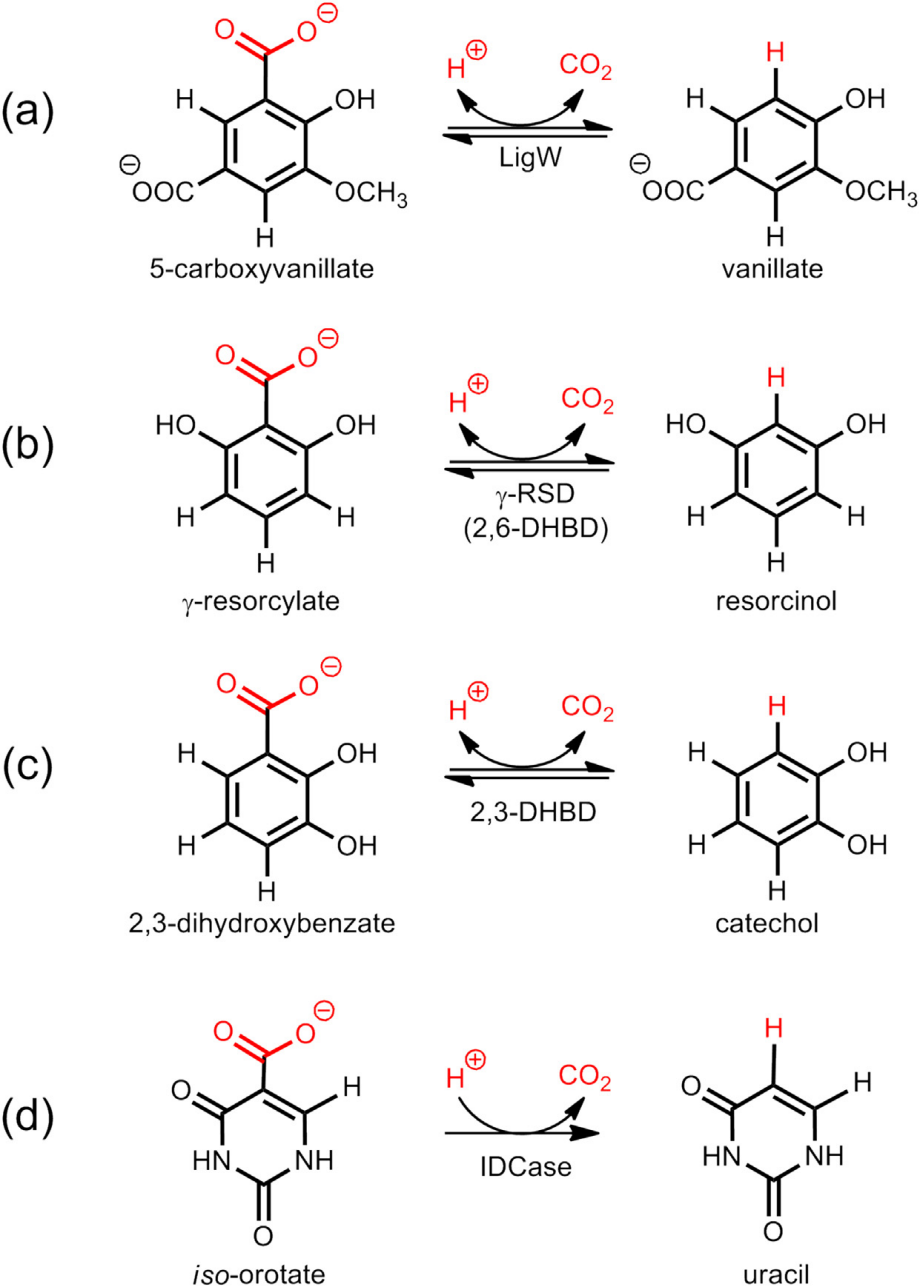
Reactions catalyzed by metal-dependent decarboxylases discussed in the present review: (a) 5-carboxyvanillate decarboxylase (LigW), (b) γ-resorcylate decarboxylase (γ-RSD, also called 2,6‐dihydroxybenzoic acid decarboxylase, 2,6-DHBD), (c) 2,3-dihydrobenzoic acid decarboxylase (2,3-DHBD), and (d) *iso*-orotate decarboxylase (IDCase).

Some of the well-studied non-oxidative metal-dependent decarboxylases are 5‐carboxyvanillate decarboxylase (LigW), γ‐resorcylate decarboxylase (γ-RSD, also called 2,6‐dihydroxybenzoic acid decarboxylase, 2,6-DHBD), 2,3‐dihydroxybenzoic acid decarboxylase (2,3-DHBD), and *iso*-orotate decarboxylase (IDCase) (Scheme 1) [18], [19], [20], [21], [22], [23], [24], [25], [26], [27], [28], [29], [30], [31], [32], [33], [34], [35]. These all belong to the amidohydrolase superfamily (AHS), the members of which more commonly catalyze the hydrolysis of carboxylate and phosphate esters [36]. Oxidative metal-dependent decarboxylases, such as, *e.g.*, coproporphyrin dehydrogenase ([Fe-S]-dependent) [37], [38], coproheme decarboxylase (Fe-dependent) [39], [40], and oxalate decarboxylase (Mn-dependent) [41], constitute another class of decarboxylases and will not be discussed here.

Interestingly, Zn was initially proposed to be the divalent metal ion commonly adopted by AHS decarboxylases [33], [35], [42], but Mn was recently determined to be the catalytic metal in LigW [43], γ-RSD [44] and IDCase [45]. 2,3-DHBD has been shown to be active with both Mn and Mg [46].

Crystal structures have been reported for a number of AHS metal-dependent decarboxylases, including the above-mentioned LigW [43], γ-RSD [33], [44], IDCase [45], and 2,3-DHBD [46]. Comparisons revealed that they have significant structural similarities, in particular the characteristic (β/α)_8_ TIM‐barrel fold and the overall active site architectures.

Detailed understanding of the mechanisms of action of these enzymes is very valuable for the development and improvement of their industrial applications. To this end, quantum chemical calculations have become a very important tool in this endeavor. In particular, the so-called cluster approach has been successfully used to study the mechanisms and selectivities of a wide range of enzymes [47], [48], [49], [50], [51], [52], [53].

We have in recent years employed this technique to uncover the reaction mechanisms of the four AHS metal-dependent decarboxylases mentioned above: LigW [54], γ-RSD [44], 2,3-DHBD [46], and IDCase [45]. In this mini-review, we summarize the results of these computational studies, all of which were published in combination with experiments. For each enzyme, the reaction mechanism obtained on the basis of the calculations will be discussed, and also the previously proposed mechanisms that turned out to be energetically unfavored will be mentioned. The new insights in terms of metal identity and substrate specificity will be described. In the interest of space, we will not discuss much the biological backgrounds of these enzymes. First, however, we will briefly describe the adopted computational methodology without going into much technical details. The interested reader is referred to some of the recent reviews on the topic [47], [48], [49], [50], [51], [52], [53].

## 2 Computational methodology

The quantum chemical cluster approach has been used to elucidate enzymatic reaction mechanism for more than 20 years. In this technique, an active site model consisting of a limited, but well-chosen part of the enzyme is designed on the basis of the crystal structures. The rest of the enzyme is truncated away, typically at C–C single bonds, and hydrogens are used to saturate the truncated bonds. The missing enzyme surrounding is modeled by a simple homogeneous polarizable medium, usually with a dielectric constant of ε = 4. The choice of ε is less critical, as systematic studies have demonstrated that the solvation effect saturates quite rapidly with the increasing model size [55], [56], [57], [58]. To maintain the overall structure of the active site model resembling the crystal structure, the atoms where the truncation is made are usually kept fixed to their crystallographic positions in the calculations.

Active site models consist today typically of 200–300 atoms. With this size it has been possible to solve a large number of diverse mechanistic problems. The size has also been shown to be sufficient to represent the chiral environment of the active site, making the approach a useful tool in the investigation of enzymatic enantioselectivity [47].

The quantum chemical electronic structure method used in the cluster approach has almost exclusively been density functional theory (DFT), in particular the hybrid B3LYP functional, which in recent years has been augmented with an empirical dispersion correction (i.e. B3LYP-D) [59], [60], [61], [62].

Mechanistic studies with the cluster approach usually start with the enzyme-substrate complex. Substrate binding and product release events are not possible to treat accurately using this technique. Since focus is on the chemical steps of the mechanisms, entropy effects are usually ignored, as they are rather small in this part of the reactions [63], [64], [65], [66], [67], [68]. However, for reaction involving binding or release of a gas molecule during the chemical steps, like the decarboxylation reactions discussed in this paper, entropy effects can be sizable and have to be taken into account. A simple way is to estimate the entropy to be equal to the translational entropy of the free CO_2_ molecule, which is ca. 11 kcal/mol. This approximation has been applied in all the studies presented here and has been shown to yield satisfactory results [44], [45], [46], [54].

## 3 5-Carboxyvanillate decarboxylase (LigW)

**Fig. 1:**
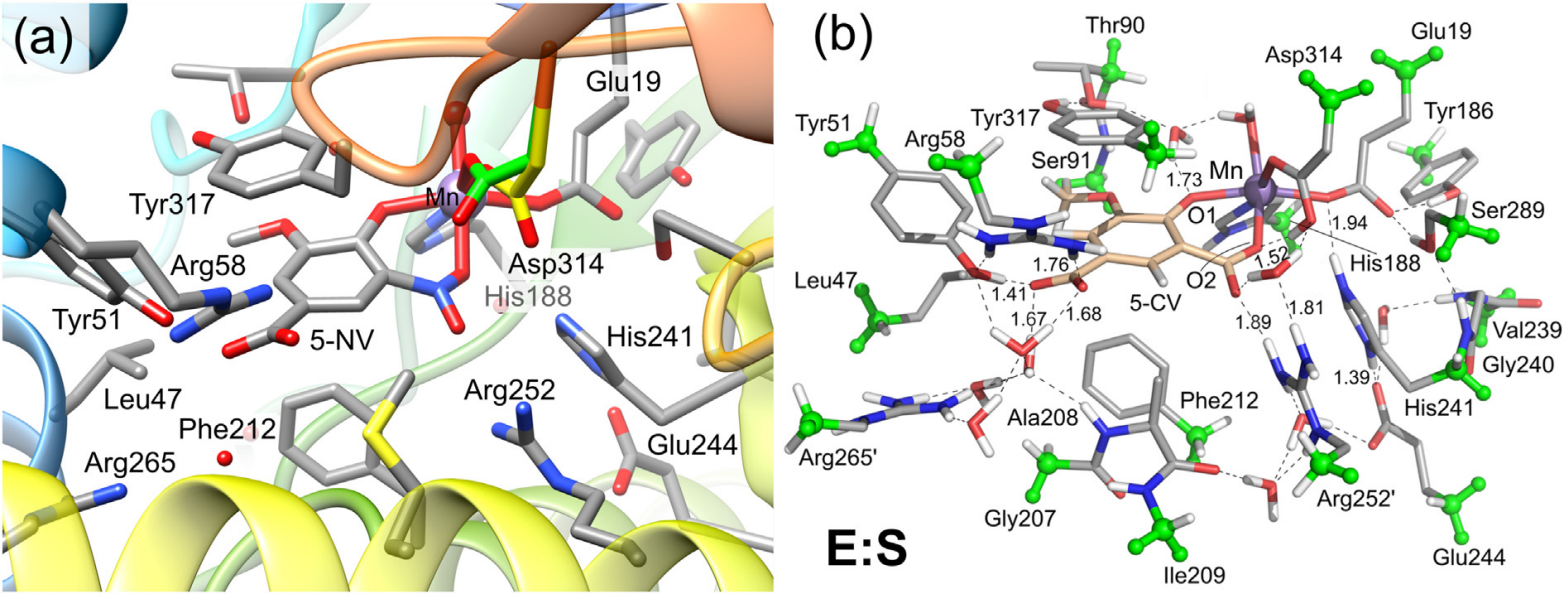
(a) Active site structure of LigW in complex with the substrate analogue 5-nitrovanillate (5-NV) (PDB: 4QRN). (b) Active site model in complex with 5-carboxyvanillate (5-CV) used in the quantum chemical calculations. Atom color scheme in this and the rest of the figures (unless stated otherwise): C grey; N blue; O red; H white. Atoms fixed during the geometry optimization are highlighted in green. Adapted from reference [54]. Copyright 2017, American Chemical Society. (For interpretation of the references to color in this figure legend, the reader is referred to the web version of this article.)

The first case to be discussed here is LigW, which catalyzes the regioselective decarboxylation of 5-carboxyvanillate (5-CV) to 3-methoxy-4-hydroxybenzoate (Scheme 1a) in the degradation pathway of lignin [69], [70]. LigW requires Mn^2+^ for the catalytic activity [43], and the high-resolution crystal structure in complex with the substrate analogue 5-nitrovanillate (5-NV) showed that the metal is coordinated by Glu19, His188, Asp314, 5-NV, and a water molecule (PDB: 4QRN) [43]. Both the hydroxyl and the nitro groups of the substrate analogue are coordinated to the metal ion, and, interestingly, 5-NV was found to have a distorted conformation with the nitro substituent being bent out of the plane of the phenyl ring (Fig. 1a). The quantum chemical calculations using an active site model designed from this structure reproduced very well the observed distortion for the inhibitor [54]. Importantly, the calculations showed that also the natural substrate (5-CV) is bent once it binds to the active site (Fig. 1b).

**Scheme 2:**
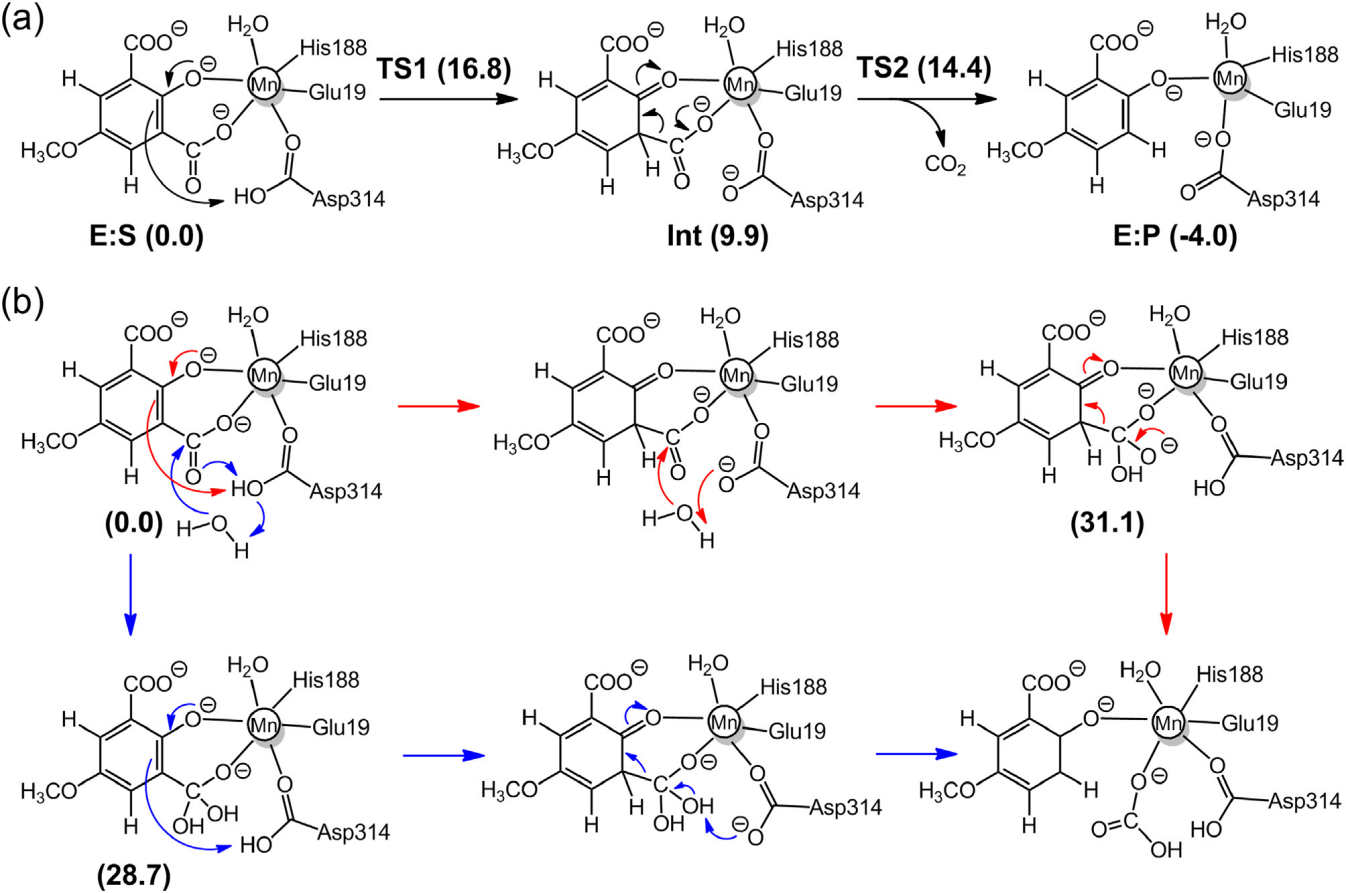
(a) Reaction mechanism for LigW proposed on the basis of the calculations. (b) Previously-proposed mechanisms (producing HCO_3_¯ as the initial product) that turned out to be associated with high energies. Calculated energies of the intermediates and transition states relative to **E:S** are given in kcal/mol. Adapted from reference [54]. Copyright 2017, American Chemical Society.

**Fig. 2:**
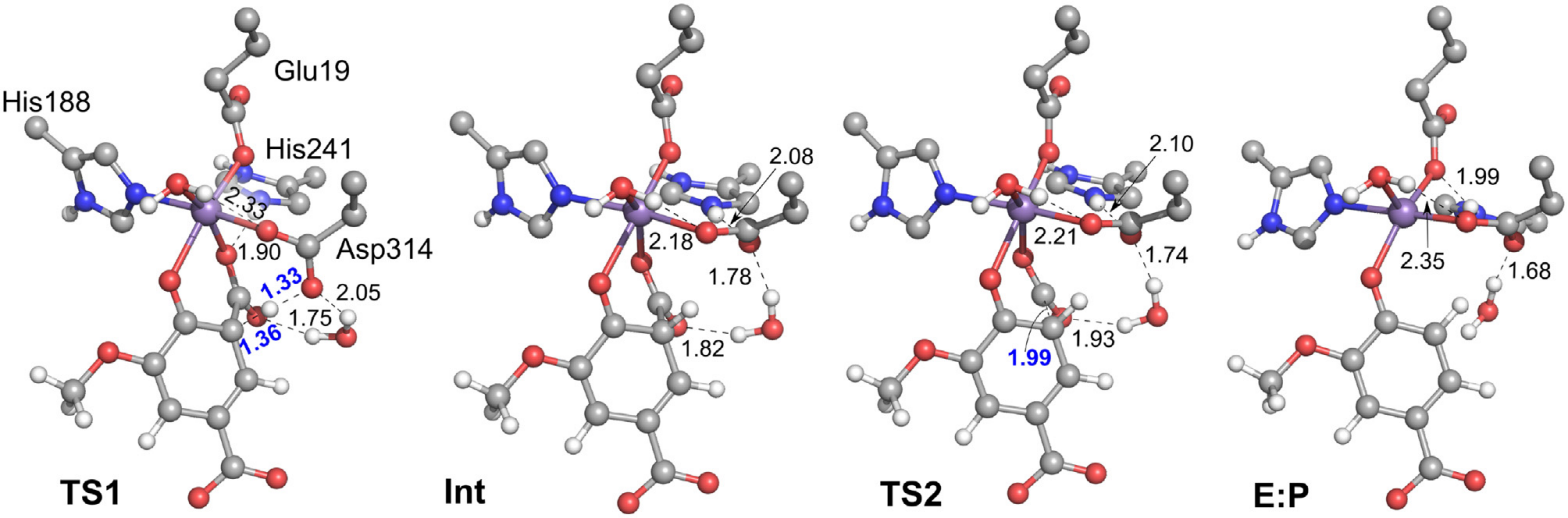
Optimized structures for the intermediates and transition states in the proposed mechanism for LigW. For clarity, only a small part of the model employed in the calculations is shown here. Selected distances are given in Å. Adapted from reference [54]. Copyright 2017, American Chemical Society.

The mechanistic investigations using the active site model shown in Fig. 1b suggested a two-step reaction mechanism for LigW, involving first a protonation of the substrate by Asp314 and subsequently a C–C bond cleavage (see Scheme 2a for mechanism and Fig. 2 for optimized structures) [54]. The proton transfer was found to be rate-limiting, with a calculated barrier of 16.8 kcal/mol, both results in excellent agreement with experimental data [43]. Namely, a kinetic isotope effect was observed experimentally amounting to 4.6, showing that the proton transfer step is rate-limiting, and the *k_cat_* value was measured to be 27 s^−1^, which corresponds to a barrier of ~16 kcal/mol.

In the optimized structures of all the intermediates and transition states along the reaction pathway, a π–π interaction is seen between the side chain of Tyr317 and the aromatic ring of the substrate. Calculations on Tyr317Ala mutant were performed, and the barrier increased by 3.6 kcal/mol compared to the wild-type enzyme. This confirms the role of Tyr317 in the catalysis, in line with the experimental results showing that this mutation lowers the activity of the enzyme by 4 orders of magnitude.[43] This result lends further support to the mechanism proposed on the basis of the calculations. The mechanism shown in Scheme 2a was subsequently corroborated by another computational study using a small active site model and two quantum mechanics/molecular mechanics (QM/MM) models [71].

It has been suggested that HCO_3_¯ rather than CO_2_ could be the initial product produced in many enzymatic decarboxylation reactions [72], [73]. This hypothesis could be examined for LigW using the quantum chemical calculations [54]. The key to form HCO_3_¯ is the formation of an intermediate with a hydrated carboxylate group, which can be formed by the attack of a water molecule at the carboxylate carbon before or after the proton transfer from Asp314 to the substrate (Scheme 2b). The two pathways were evaluated by calculating the energies of the corresponding intermediates. It turned out both are associated with prohibitively high energies, ca. 30 kcal/mol higher than the enzyme-substrate complex (Scheme 2b), and these mechanistic possibilities could thus be ruled out. This finding was corroborated by membrane inlet mass spectrometry (MIMS) experiments [54]. Moreover, the same conclusion had previously been obtained for another decarboxylase, phenolic acid decarboxylase [74].

The results indicate thus that CO_2_ is the direct carboxylating agent in the reverse carboxylation reaction, and it is therefore conceivable that CO_2_ gas can be used for the carboxylation instead of HCO_3_¯ buffer. Indeed, Plasch et al. showed that resorcinol could be carboxylated directly by 2,3-DHBD and salicylic acid decarboxylase using pressurized CO_2_, with up to 68% conversion [75].

## 4 γ-Resorcylate decarboxylase (γ-RSD)

**Fig. 3:**
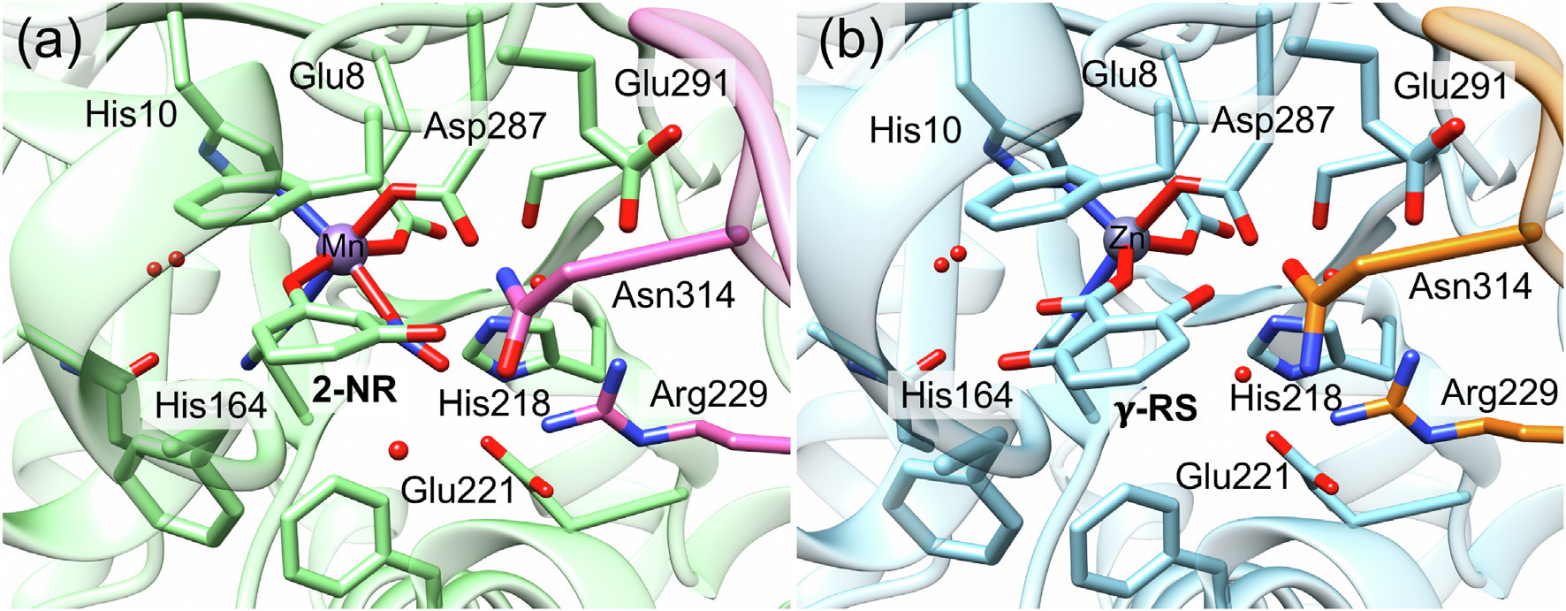
Active site structures of (a) Mn-containing γ-RSD from Polaromonas sp. JS666 (PDB: 4QRO) and (b) Zn-containing γ-RSD from Rhizobium sp. MTP-10005 (PDB: 2DVU). In this figure, the carbon atoms are displayed in light green and pink in (a), and light blue and orange in (b), for better clarity. (For interpretation of the references to color in this figure legend, the reader is referred to the web version of this article.)

γ-Resorcylate decarboxylase catalyzes the decarboxylation of γ-resorcylate (γ-RS) to resorcinol, as shown in Scheme 1b [76], [77]. The enzyme has a relatively high conversion and a wide substrate scope in the reverse carboxylation activity [22], [23], [24], [25], [26], [27]. Crystal structures have been solved for γ-RSD from *Rhizobium* sp. MTP-10005 [33], and more recently from *Polaromonas* sp. JS666 [44]. γ-RSD from *Polaromonas* sp. JS666 was proposed to be Mn-dependent (Fig. 3a), and the crystal structure with the substrate analogue 2-nitroresorcinol (2-NR) showed the metal ion coordinated by Glu8, His10, His164, Asp287 and 2-NR (PDB: 4QRO) [44]. The binding mode of 2-NR is similar to the substrate binding in LigW, with both the hydroxyl and the carboxylate groups coordinated to the metal in a bidentate fashion [43].

**Scheme 3:**
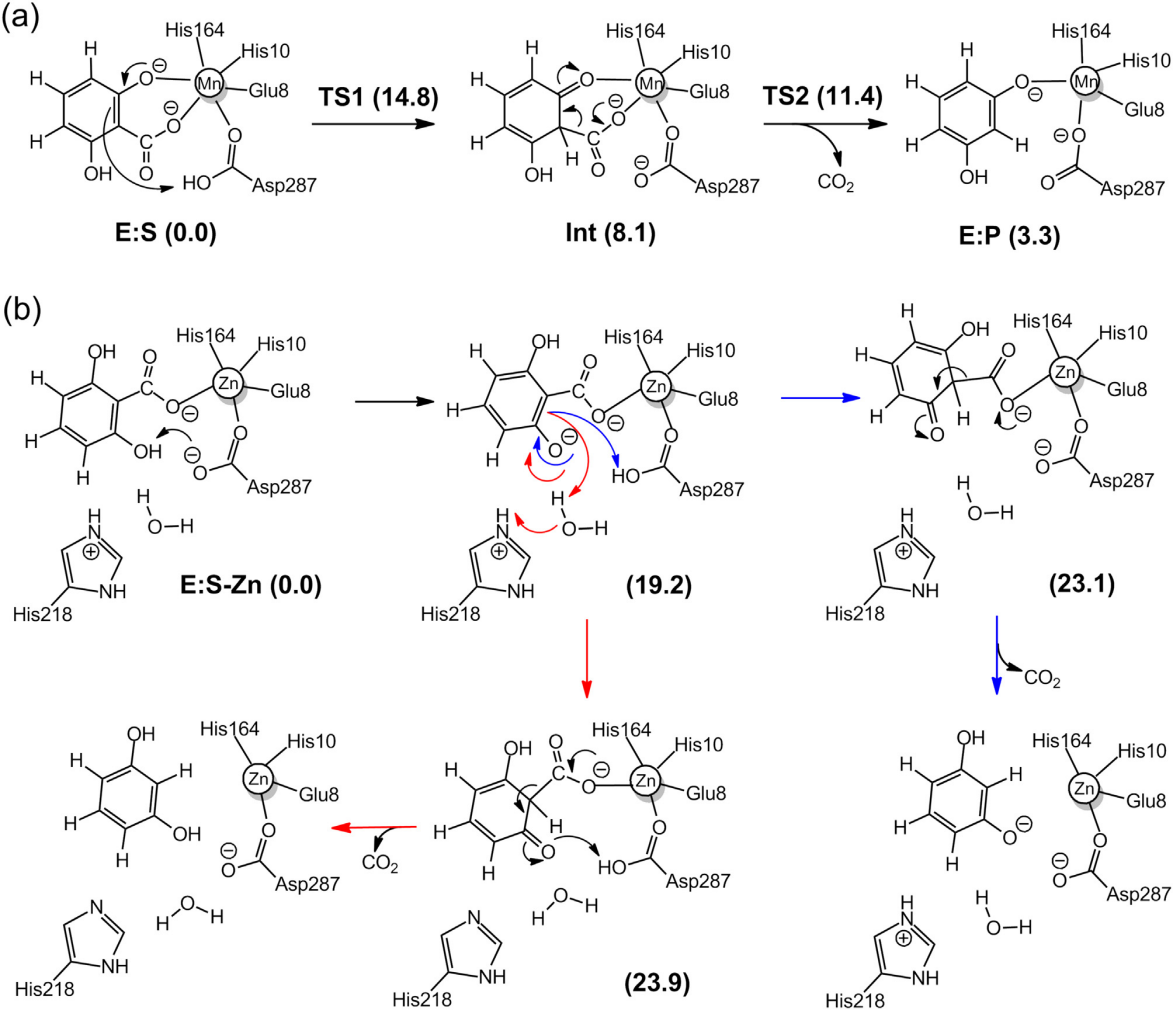
(a) Proposed reaction mechanism for the Mn-containing γ-RSD. (b) Previously-suggested mechanisms for the Zn-containing γ-RSD. Calculated energies of intermediates and transition states relative to the respective enzyme-substrate complex are given in kcal/mol. Adapted from reference [44]. Copyright 2018, American Chemical Society.

Calculations on the Mn-containing γ-RSD from *Polaromonas* sp. JS666 showed that this enzyme has the same mechanism as LigW, involving a proton transfer from Asp287 to the substrate and a subsequent C–C bond cleavage (Scheme 3a) [44]. The geometries of the intermediates and TSs, as well as the energies are also very similar to those obtained for LigW. For γ-RSD, the protonation step is rate-limiting with a barrier of 14.8 kcal/mol, and the C–C bond cleavage has a barrier of 11.4 kcal/mol.

γ-RSD from *Rhizobium* sp. MTP-10005 was proposed to contain a Zn ion instead [33]. In the obtained structure of the wild-type γ-RSD in complex with the substrate (PDB: 2DVU), the metal was observed to be coordinated by γ-RS and the same residues as those in the Mn-containing enzyme discussed above (Fig. 3) [33]. However, in this structure γ-RS is bound monodentately with only one oxygen of the carboxylate group coordinating to the metal, and none of the hydroxyl groups (Fig. 3b).

Based on the substrate-bound structure, the reaction of the Zn-containing γ-RSD had been proposed to be initiated by the deprotonation of the hydroxyl group of the substrate with Asp287 being the general base. This is then followed by the protonation of the carbon and a subsequent C–C bond cleavage (Scheme 3b) [33]. The calculations could not give support to this mechanistic scenario, because the calculated energies of the 2,4-dienone intermediates in the suggested pathways are prohibitively high, more than 20 kcal/mol relative to the corresponding enzyme-substrate complex. The monodentate binding mode was thus concluded to be unproductive for the Zn-containing γ-RSD. The same conclusion was also obtained for the Mn-containing γ-RSD [44].

## 5 2,3‐Dihydroxybenzoic acid decarboxylase (2,3-DHBD)

2,3‐dihydroxybenoic acid decarboxylase from *Aspergillus oryzae* catalyzes the transformation of 2,3‐dihydroxybenzoic acid to catechol (Scheme 1c). The nature of the metal ion in 2,3-DHBD was investigated and the reaction mechanism was addressed in a joint experimental-computational study [46].

**Fig. 4:**
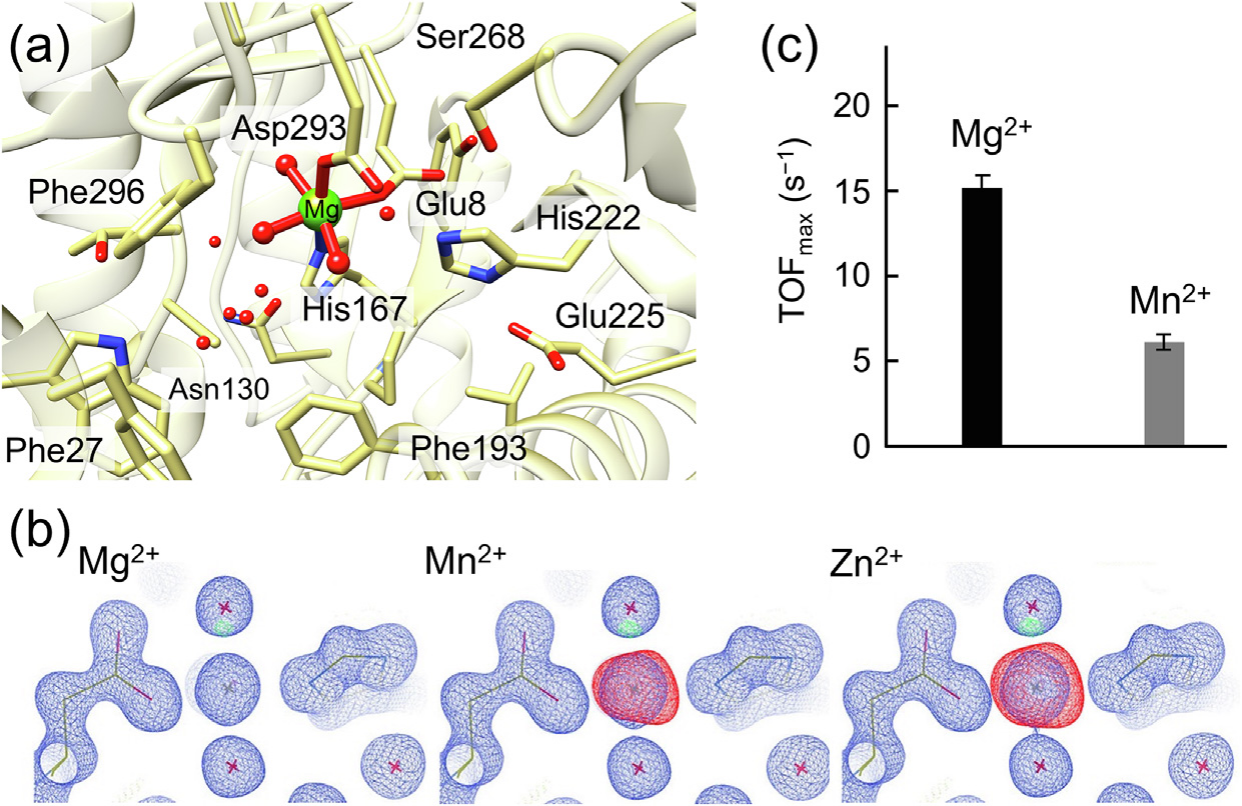
(a) Active site structure of 2,3-DHBD from Aspergillus oryzae (PDB: 7A19). (b) Electron density map of models with Mg^2+^, Mn^2+^ or Zn^2+^ (in blue) contoured at 1.5 rmsd, and difference map drawn at 5 rmsd showing the excess of model electrons for Mn and Zn (in red). (c) Steady‐state turnover frequency of 2,3‐DHBD with either Mn or Mg. The carbon atoms are displayed in khaki for better clarity. Adapted from reference [46]. (For interpretation of the references to color in this figure legend, the reader is referred to the web version of this article.)

A high-resolution crystal structure could be obtained for the ligand-free form of the enzyme, in which the metal was observed with a well-defined electron density, coordinated by Glu8, His167, Asp293 and three water molecules (Fig. 4a) [46]. Different divalent metals were considered in the interpretation of this electron density. Both Mn and Zn overestimated the density, and it was surprisingly found that Mg gave an excellent fit (Fig. 4b). Further elemental mass spectroscopy and activity measurements showed that 2,3-DHBD is catalytically active with Mn, similarly to LigW and γ-RSD, but displays a significantly improved activity with Mg (Fig. 4c) [46]. Furthermore, it was shown that the enzyme from *Aspergillus oryzae* does not accept Zn, in contrast to the enzyme from *Fusarium oxysporum* [42].

**Scheme 4:**
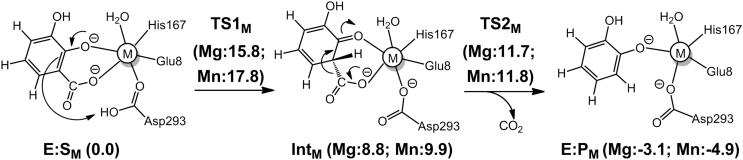
Proposed reaction mechanism for 2,3-DHBD. The calculated energies of the intermediates and transition states relative to the corresponding enzyme-substrate complex are given in kcal/mol.

Quantum chemical calculations demonstrated that 2,3-DHBD from *Aspergillus oryzae* follows a similar mechanism as that proposed for LigW and γ-RSD [46]. Again, the reaction starts with the rate-limiting proton transfer from a general acid (Asp293), followed by a C–C bond cleavage to yield the products (Scheme 4). The energy profiles of 2,3-DHBD were calculated with active site models using either Mg and Mn ions, and both cases displayed similar energies as those obtained for LigW and γ-RSD [46]. The overall barrier of the Mn-enzyme was calculated to be 2 kcal/mol higher than that of the Mg-enzyme (15.8 kcal/mol *vs* 17.8 kcal/mol), in agreement with the experimental trend (Fig. 4c).

The substrate binding mode was also investigated [46]. The monodentate binding of 2,3-dihydroxybenzate was calculated to be 2.8 and 1.8 kcal/mol higher than the bidentate mode for Mg- and Mn-enzymes, respectively, showing that the productive bidentate mode is more favored. This is different from the case of the Mn-dependent γ-RSD discussed above, for which the two binding modes had almost identical energies [44].

## 6 *Iso*-orotate decarboxylase (IDCase)

The final case study discussed here is *iso*-orotate decarboxylase, which differs from the above decarboxylases in that the substrate is a heterocyclic compound. IDCase is involved in the thymidine salvage pathway, catalyzing the decarboxylation of *iso*-orotate (5-carboxyuracil, 5caU) to uracil, as shown in Scheme 1d [78], [79].

**Scheme 5:**
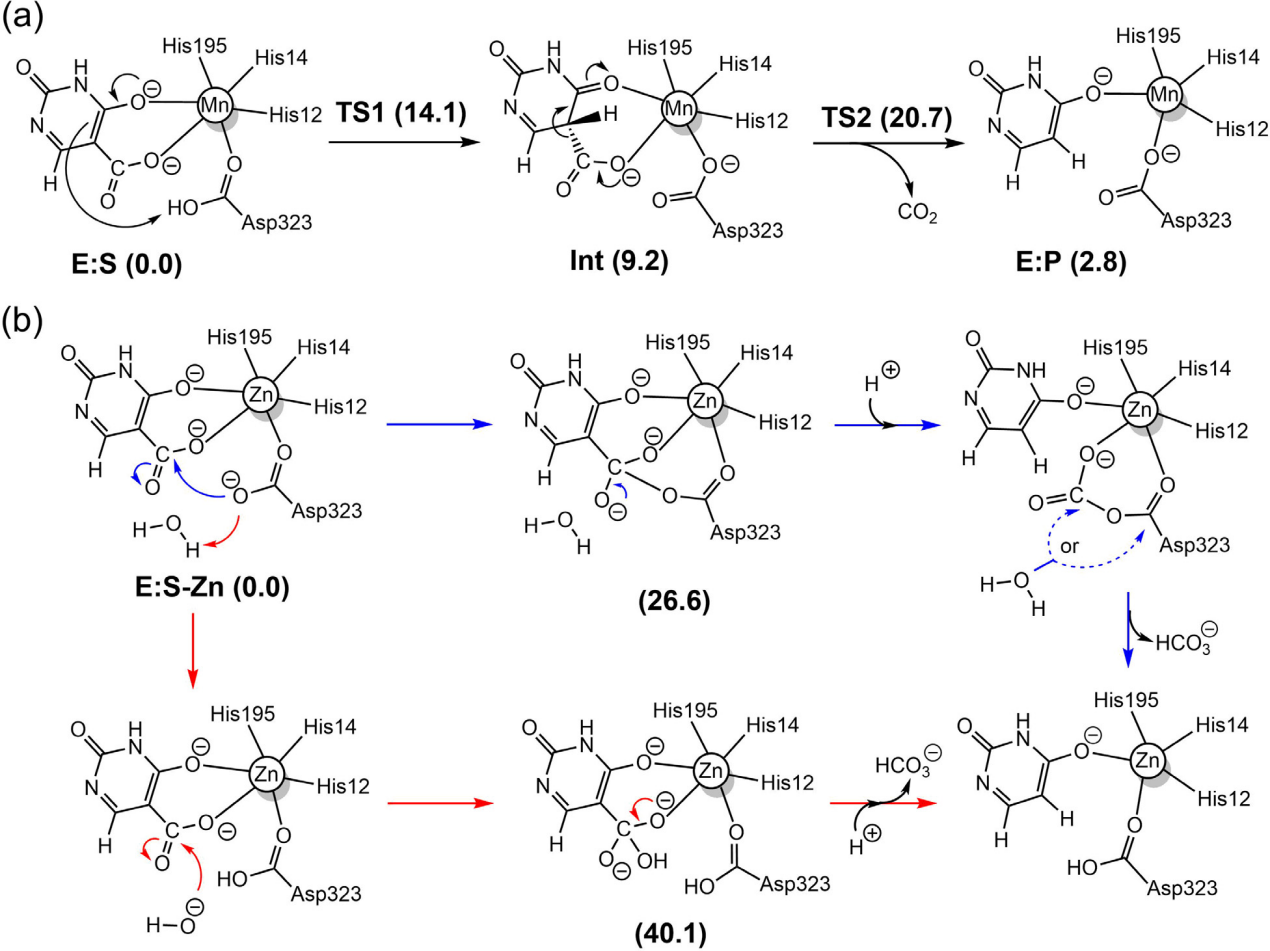
(a) Proposed reaction mechanism for the Mn-enzyme of IDCase and (b) the previously proposed mechanisms for the assumed Zn-enzyme of IDCase. The calculated energies of the intermediates and transition states relative to the corresponding enzyme-substrate complexes are given in kcal/mol. Adapted from reference [45].

**Fig. 5:**
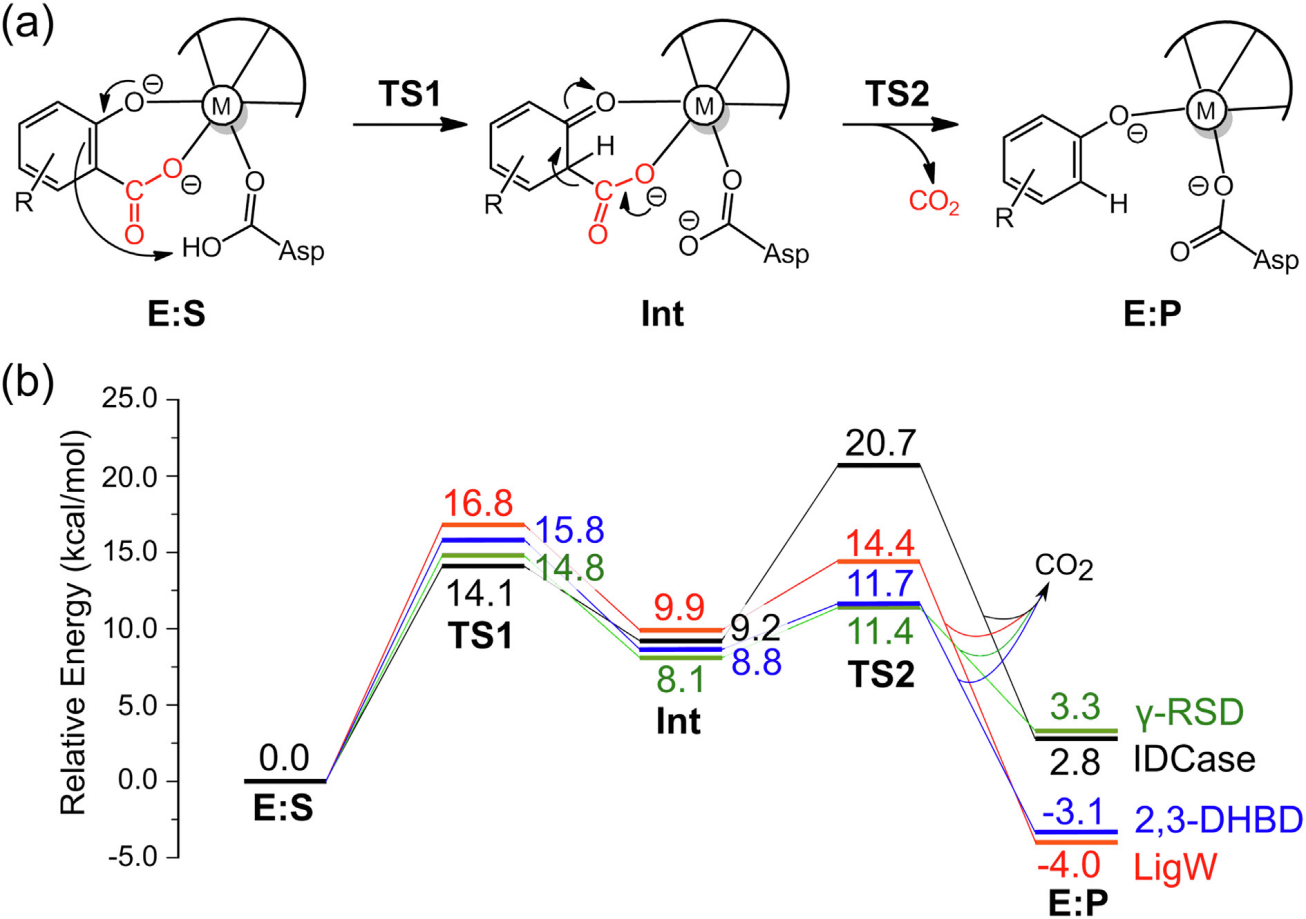
(a) A general mechanism suggested for metal-dependent decarboxylases. (b) Calculated energy profiles of the different enzymes discussed in the present review.

A number of crystal structures of IDCase have been solved [35]. The structure of the Asp323Asn variant in complex with substrate 5caU (PDB: 4LAM) shows that the divalent metal is coordinated by Asp323 (Asn in the mutant), three histidine residues (His12, His14 and His195), and 5caU. The metal in IDCase was previously assumed to be Zn. However, the substrate was found to bind in a bidentate fashion, with both the hydroxyl and carboxylate groups coordinated to the metal, which is the same binding mode that had been established for Mn-dependent LigW and γ-RSD [44], [54]. This suggested that IDCase might also be Mn-dependent, and indeed, ICPMS/MS measurements could unambiguously demonstrate that this is the case [45]. Also the computational mechanistic investigation of the Mn-dependent IDCase showed that it follows exactly the same mechanism as LigW and γ-RSD [45]. Protonation of the substrate takes first place with Asp323 as the general acid group, and then C–C bond cleavage occurs, generating CO_2_ and uracil (Scheme 5a). The energy profile showed some interesting differences compared to the other cases (Fig. 5). The C–C bond cleavage in IDCase was found to be rate-limiting, with the barrier being ca 7 kcal/mol higher than the protonation step.

Calculations were also performed assuming that IDCase uses Zn as the metal cofactor, as originally supposed [35]. Two proposed mechanisms were examined (Scheme 5b) and both were found to be associated with prohibitively high energies and could thus be ruled out [45]. In both mechanisms, HCO_3_¯ is assumed to be generated as the initial product, and the high energies obtained show again that this hypothesis is not a viable option.

**Scheme 6:**
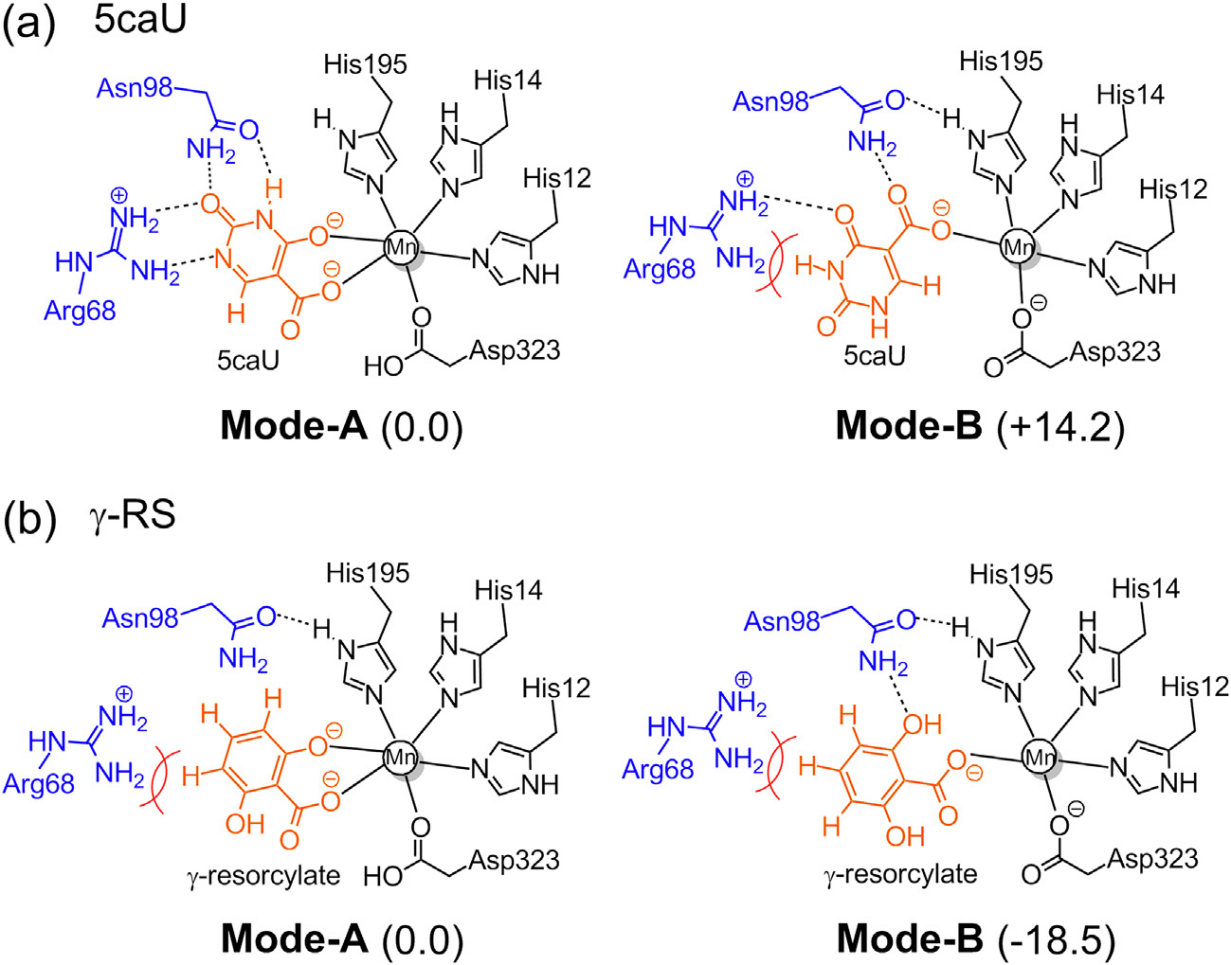
Mono- and bidentate binding modes of 5caU and γ-RS in the Mn-dependent IDCase. Relative energies are given in kcal/mol. Adapted from reference [45].

In contrast to the other enzymes discussed above, IDCase exhibits a rather narrow substrate scope. It is not active in the decarboxylation of phenolic carboxylic acids, nor in the carboxylation of uracil, pyrimidine derivatives or phenols [45]. An active site comparison of IDCase with LigW and γ-RSD helped to obtain a structural basis for the substrate specificities of these enzymes. The low substrate tolerance of IDCase could be explained by some key interactions between the natural substrate and the surrounding active site residues, such as Arg68 and Asn98. Accordingly, suitable mutations of these identified residues could lead to the expansion of the substrate scope of IDCase. Comparison of the energetics of the bidentate (**Mode-A**) and monodentate (**Mode-B**) binding modes for the 5caU natural substrate compared to γ-RS, which is not accepted by IDCase, provided supports to this hypothesis (Scheme 6) [45]. For 5caU, the productive **Mode-A** was found to be much more preferred than the unproductive **Mode-B** (by 14.2 kcal/mol), while in the case of γ-RS, **Mode-B** is much more favorable, by 18.5 kcal/mol.

## 7 Conclusions

We have in this mini-review summarized our computational studies on four metal-dependent decarboxylases from the amidohydrolase superfamily. On the basis of detailed quantum chemical calculations employing large active site models, important insights could be obtained regarding the reaction mechanisms, metal identities, and substrate specificities.

A common reaction mechanism could be established for all these enzymes, as shown in Fig. 5. The reaction starts by the substrate binding bidentately to the divalent metal ion. Although it is possible for the substrate to bind monodentately in some enzymes, this binding mode is not catalytically productive, as it leads to high barriers. The reaction proceeds by a protonation of the carbon by an active site Asp residue, followed by a final C–C bond cleavage, releasing CO_2_. We believe that this simple mechanistic scheme is rather general and has a bearing on other metal-dependent decarboxylases.

Taken together, the discussed cases demonstrate clearly that the adopted cluster approach is indeed a very productive tool in mechanistic studies of enzyme reactions, adding further to the experience gained in this field in the last two decades. The calculations can rationalize experimental observations in terms of selectivity and substrate specificity. The current results show moreover that the obtained energy profiles can be used to indirectly assign metal identity in the enzymatic reactions. It is therefore not difficult to predict that more studies of this kind will be produced in the future.

## Declaration of Competing Interest

The authors declare that they have no known competing financial interests or personal relationships that could have appeared to influence the work reported in this paper.
